# Speech speed in audiovisual resources in the learning process of university students: a scoping review

**DOI:** 10.1590/2317-1782/e20240326en

**Published:** 2026-01-30

**Authors:** Kelly da Silva, Pablo Jordão Alcantra Cruz, Nayara Farias Costa Santos, Flávia Vanessa Menezes de Jesus, Mara Behlau, Anna Alice Almeida, Raphaela Barroso Guedes-Granzotti

**Affiliations:** 1 Programa de Pós-graduação em Ciências Aplicadas à Saúde, Universidade Federal de Sergipe – UFS - Lagarto (SE), Brasil.; 2 Departamento de Fonoaudiologia, Universidade Federal de Sergipe – UFS - Lagarto (SE), Brasil.; 3 Centro de Estudos da Voz – CEV - São Paulo (SP), Brasil.; 4 Departamento de Fonoaudiologia, Universidade Federal da Paraíba – UFPB - João Pessoa (PB), Brasil.; 5 Departamento de Fonoaudiologia, Universidade Federal de Sergipe – UFS - São Cristóvão (SE), Brasil.

**Keywords:** Processing Speed, Learning, University Students, Speech, Comprehension

## Abstract

**Purpose:**

This study aimed to map studies that investigated the influence of speech rate on the reproduction of audiovisual resources in university students' learning using a Scope review.

**Research strategies:**

The PCC strategy was used where P- University students, C- Increased or decreased speech rate, C- Learning. The searches were conducted using the SciELO, Lilacs, PubMed, Scopus, Web of Science, and Google Scholar databases.

**Selection criteria:**

Scientific articles published in the databases above with a target audience of university students were included; works that compared average speech rate with increased or decreased speed and had learning-related skills as the study response.

**Data analysis:**

The Joanna Briggs Institute checklists were used for methodological quality assessment.

**Results:**

We found that four articles observed an improvement in learning performance when the audiovisual resource was presented at a speed of up to 2x, three did not observe a difference and two studies observed a worsening in the skills involved in learning.The results indicate a low risk of bias in most studies included.

**Conclusion:**

A study indicated improved learning in audiovisuals at a speed slower than 1x. It was observed that reproduction speed rates of audiovisual resources impact university students' learning process. Rates greater than 2x limit the learning process. Rates within the normal range (between 1x and 1.75x) are more favorable for capturing the content.

## INTRODUCTION

In the modern world, the demand for urgency and the efficient and productive use of time is a constant challenge, making it a valuable commodity^(1)^. In this context, in the quest for knowledge, students have been using various tools to facilitate learning^(2)^. Recorded classes, remote lessons, and educational audio are currently used to acquire information^(3)^. Students have resorted to accelerating the playback of audiovisual resources to save time and potentially optimize their educational experiences^(4)^.

The flexibility in delivering content has expanded the resources for learning, studying, updating, and staying informed. These resources offer functions such as speed control and the ability to resume and advance videos, allowing students to manipulate their time to verify their learning. Some contribute to reducing the time spent, while others enhance comprehension and learning^(5,6)^.

Learning can be understood as the process of acquiring new information that will be stored in memory, enabling individuals to guide their thinking and behavior. Memory, in turn, refers to the selective storage of this information, allowing it to be retrieved—consciously or unconsciously—whenever needed. In this sense, learning can be seen as a set of behaviors that support the neurobiological and neuropsychological processes of memory. Given the close relationship between the concepts of learning and memory, it is common for these terms to be used interchangeably in various contexts^(7)^.

However, the literature does not present a consensus on the effects of using this tool on human cognitive processes, especially on executive functions such as attention, memory, and various aspects of central auditory processing related to the comprehension and learning of the received message. This raises practical questions: Does the process of quickly obtaining information put the subject in control of knowledge? Can the speed at which we receive information hinder the acquisition and consolidation of information? Does our memory have sufficient capacity to absorb many stimuli in a reduced time? This study aims to provide answers to these questions and equip university students with the knowledge to make informed decisions about the speech rate in the playback of audiovisual resources, potentially enhancing their learning process. Therefore, the objective of this review was to map the syntheses of evidence on a speech rate that interferes with learning when used by university students.

## METHODS

### Study design

This study is a scoping review, a methodological approach recommended by PRISMA-ScR^(8)^ and the Joanna Briggs Institute Manual for Evidence Synthesis for Scoping Reviews^(9)^. The research was registered on the Open Science Framework – OSF platform (10.17605/OSF.IO/X2DCA). The research strategy involved several stages: formulating the research question, defining inclusion and exclusion criteria, conducting a systematic search for relevant studies, selecting studies based on their titles and abstracts, reading the full texts of the selected studies, extracting data, presenting the results, and analyzing the risk of bias.

### Inclusion criteria

The eligibility criteria were defined using the PCC format (Participants, Concept, Context). Eligibility for the review was as follows: Participants - University students; Concept - Increased or decreased speech rate; Context - Learning.

Scientific articles published in the databases above with a target audience of university students were included; works that compared average speech rate with increased or decreased speed and had learning-related skills as the study response, without limitation of study design. Studies involving only a second language; studies with the following populations: elderly university students (over 60 years), with neurodivergence or deafness/hearing loss; outside the scope of our research; did not have at least one group with any speed alteration; with used learning supports, and articles introducing competitive noise in addition, Systematic reviews, opinion texts, incomplete articles, duplicated articles (only one version was considered), were excluded from the analysis.

### Research sources and search strategy

Searches were conducted in the SciElo, Lilacs, PubMed, Scopus, Web Of Science, and Google Scholar databases (the first 100 articles) from September 11 to 13, 2023. The search was updated on March 8, 2025, with year filters from 2023 to 2025, except for the Google Scholar database, where the top 100 reported studies were reanalyzed. The search strategy was developed based on specific keywords considering the PCC elements. Relevant keywords and vocabularies controlled in Medical Subject Headings and EMTREE were used to search each concept of interest (Appendix A) and to elaborate the final search strategies. References were managed, and duplicate studies were removed using Rayyan software

### Selection

Rayyan software was used, which helps in organizing scoping reviews. The first selection stage, reading titles and abstracts, involved identifying eligible studies conducted by two independent reviewers who, without direct interaction, simultaneously performed the selection following the pre-established search strategies. This stage involved two reviewers, with a third reviewer participating only in case of disagreements. After this stage, the full text was read, and the studies meeting all eligibility criteria were systematically analyzed for information regarding the title, authors, publication year, country, study objective, research type, sample size, outcome assessment method, and main results. These data were collected according to a pre-elaborated protocol by the researchers to extract maximum data from the studies and systematically organized in tables for subsequent analysis.

### Data extraction and analysis

The following data were extracted from the selected articles: authors (year), country, main objective, sample, main results. Microsoft Excel was used to compile and store the data collected.

The methodological quality of the included studies was independently assessed by two reviewers, following the Joanna Briggs Institute (JBI) protocol for Quasi-Experimental Studies^(9)^. The assessment was based on 9 questions, with possible responses being “yes,” “no,” “unclear,” or “not applicable.” The studies were classified into three levels: high, moderate, and low quality. Quality was considered high when 70% or more of the responses were “yes,” moderate for scores between 50% and 69%, and low when the score was equal to or less than 49%^(10)^.

This stage was conducted to elucidate to readers the methodological quality of the primary works that comprised this review. A figure was created in Word to illustrate the main results of this analysis.

## RESULTS

**Figure 1:**
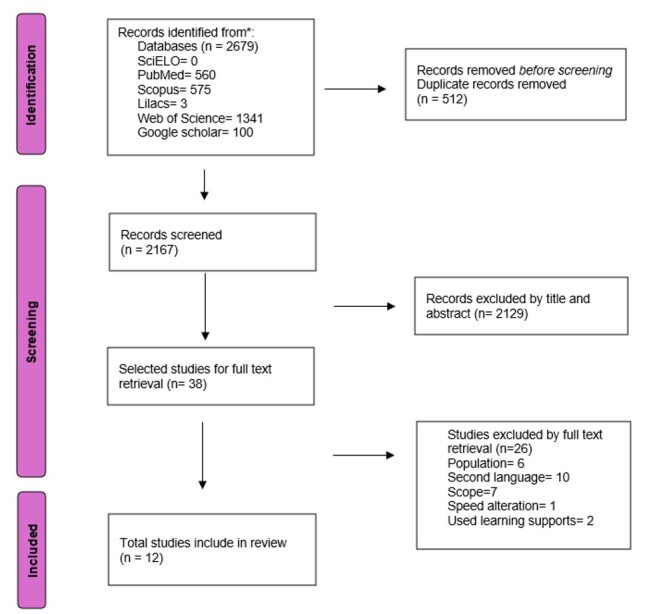
Search and selection flowchart of articles

A total of 2679 articles were identified, and twelve were included after the selection process (Figure 1).

**Figure 2:**
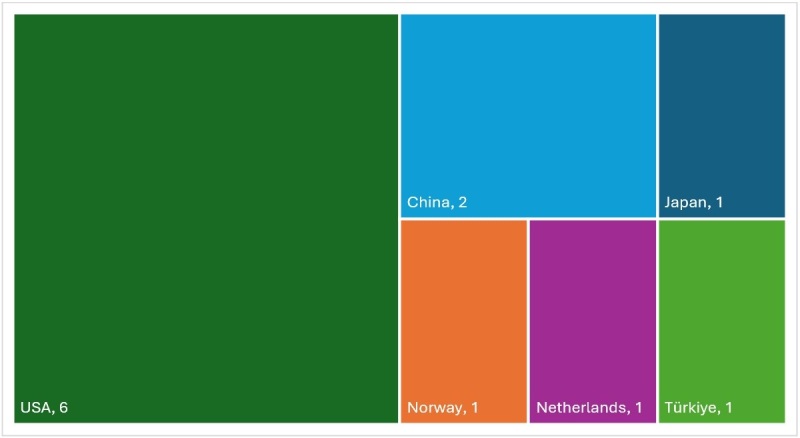
Number of articles by country

Table 1 presents the mapping of data from the twelve studies included. The studies included were published between 2017 and 2024 and in six different countries; with 50% of the studies being developed in the USA (Figure 2). We found that five articles observed an improvement in learning performance when the audiovisual resource was presented at a speed of up to 2x^(6,11,14,17,19)^, five did not observed a difference^(4,12,15,18,20 )^ and two studies observed a worsening in the skills involved in learning^(13,16)^(Table 1).

**Table 1:** Synthesis of eligible studies

Authors (year)	Country	Main Objective	Sample	Main Results
Nagahama and Morita^(11)^	Japan	Analyze content **comprehension** after video lessons with slides and instructor images at 1x, 1.5x, and 2x speeds.	59	Watching videos at 1.5x speed was more effective for learning than average speed.
Jacobson et al.^(12)^	USA	Investigate video lesson acceleration practice and **comprehension** at 1x, 2x, and 3x speeds.	49	For students practicing in video acceleration, comprehension was the same at normal and 2x speed. However, 3x acceleration reduced comprehension regardless of practice.
Song et al.^(13)^	USA	Compare average scores on written assessments after watching a recorded lecture at 1x and 1.5x speeds and analyze immediate content **retention ability**.	54	Data showed negative results at 1.5x speed compared to average speed.
Fountoukido et al.^(14)^	Netherlands	Investigate the effects of a virtual model with manipulated vocal parameters (pitch, speech rate, and prosody) on university students' **learning**.	144	Participants receiving instructions from a virtual model with higher intensity and faster speed showed more significant learning and perception of knowledge than those with lower intensity and slower speed.
Lang et al.^(6)^	USA	Analyze the relationship between watching videos at 1x or 1.25x speed and students' **learning** outcomes.	-	Students who watched accelerated content were more likely to achieve better grades. Accelerated videos resulted in students spending less time watching videos and having a significantly higher likelihood of completing more video views.
Murphy et al.^(15)^	USA	Investigate if watching lecture videos at various speeds affects **comprehension and metacognitive learning** monitoring.	106	There was no significant difference in learning between increasing speeds from 1x to 1.5x and 2x. Students had more difficulty comprehending videos played at speeds above 2.5x.
Ness et al.^(16)^	Norway	Evaluate the effects of 1x and 2x video playback speeds on **learning**.	20	Test scores were significantly lower at 2x playback speed.
Mo et al.^(17)^	China	Analyze how video playback speed affects **learning and cognitive** load when watching videos at four speeds (1x, 1.25x, 1.5x, and 2x).	76	Increased speeds of 1.25x and 1.5x positively influenced learning, varying according to students' learning abilities. High-level students performed better at 1.5x speed, while low-level students performed better at 1.25x speed. The highest learning effect was observed under medium cognitive load.
Merhavy et al.^(4)^	USA	Evaluate the associations between lecture playback speeds of 1.5x and 2x with **concentration and long-term memory retention**.	33	There is no significant difference in concentration or long-term memory retention at 1.5x compared to 2x playback speed.
Simonds et al.^(18)^	USA	The main objective of the study was to investigate the effects of instructor speech rate on student affective learning, **recall of information**, and perceptions of nonverbal immediacy, credibility, and clarity of the instructor	181	The study revealed that students perceived higher credibility and nonverbal immediacy from the instructor at the moderate speaking rate (172 wpm), as well as greater affective learning. No significant differences were found in clarity or recall between the different speaking rates.
Liu and Jia^(19)^	China	This study aims to investigate how video playback speed and the presence of pre-embedded questions affect students' performance, attention allocation, and **learning outcomes**, aiming to guide the choice of the ideal playback speed and provide insights for the interactive design of videos.	90	The study showed that pre-embedded questions increased participants' attention, and the 1.25x playback speed improved comprehension by reducing fixation duration. Both factors also positively impacted information retention, suggesting that questions and an appropriate playback speed improve students' attention and retention.
Kıyak et al.^(20)^	Türkiye	The study aimed to determine how watching lecture videos at 1x and 2x speeds affects **memory retention** in medical students	60	The study showed that the video playback speed (1x or 2x) had no significant effect on the memory retention scores of medical students, both in the immediate and delayed tests. Both groups showed a decrease in scores in the delayed test, but there was no significant difference between the speeds, suggesting that watching video lessons at double speed did not affect retention.

The results in Table 2 show the analysis of the methodological quality of the studies included in this review, according to the JBI critical appraisal tool for quasi-experimental studies^(9)^.

**Table 2:** Methodological quality of the included studies

**Study**	**Q1**	**Q2**	**Q3**	**Q4**	**Q5**	**Q6**	**Q7**	**Q8**	**Q9**	**% of yes (Methodological quality)**
Simonds et al.^(18)^	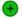	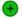	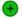	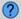	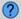	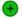	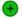	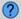	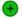	66,6% (Moderate quality)
Liu and Jia.^(19)^	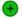	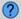	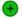	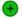	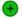	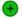	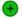	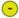	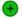	77,7% (High quality)
Kıyak et al.^(20)^	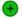	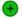	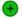	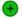	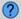	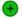	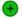	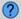	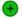	77,7% (High quality)
Jacobson et al.^(12)^	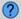	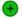	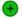	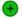	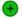	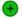	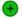	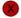	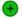	77,7% (High quality)
Nagahama and Morita^(11)^	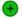	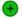	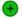	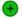	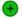	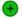	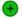	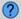	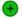	88,8% (High quality)
Fountoukidou et al.^(14)^	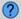	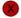	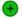	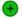	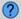	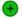	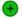	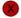	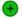	55,5% (Moderate quality)
Mo et al.^(17)^	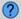	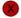	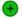	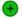	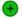	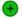	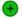	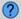	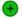	66,6% (Moderate quality)
Lang et al.^(6)^	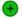	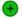	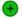	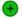	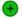	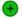	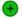	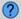	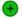	88,8% (High quality)
Murphy et al.^(15)^	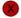	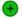	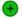	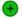	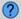	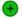	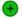	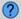	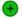	66,6% (Moderate quality)
Ness et al.^(16)^	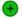	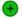	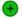	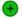	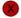	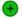	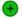	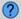	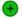	77,7% (High quality)
Merhavy et al.^(4)^	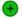	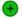	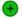	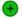	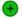	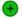	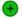	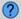	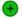	88,8% (High quality)
Song et al.^(13)^	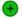	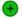	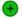	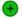	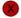	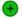	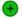	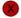	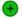	77,7% (High quality)

**Caption:** 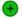 = Yes; 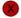 = No; 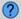 = Unclare; 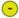 = Not applicable . Q1. Is it clear in the study what is the “cause” and what is the “effect” (i.e. there is no confusion about which variable comes first)?; Q2. Was there a control group?; Q3. Were participants included in any comparisons similar?; Q4. Were the participants included in any comparisons receiving similar treatment/care, other than the exposure or intervention of interest?; Q5. Were there multiple measurements of the outcome, both pre and post the intervention/exposure?; Q6. Were the outcomes of participants included in any comparisons measured in the same way?; Q7. Were outcomes measured in a reliable way?; Q8. Was follow-up complete and if not, were differences between groups in terms of their follow-up adequately described and analyzed?; Q9. Was appropriate statistical analysis used?

## DISCUSSION

The speed rate at which we present audiovisual resources is a crucial factor that significantly influences the learning process in students, making it a topic of significant interest and importance in the field of education and technology.

In the contemporary world, technology has emerged as a versatile and practical tool that connects people. Particularly in the context of the global pandemic, it has proven to be an essential interface, enabling remote education as a means of adaptation in the face of adverse circumstances across various areas of knowledge and cultures^(21,22)^.

Amidst this reality, the ability to control the playback speed of audiovisual content has emerged as a viable and attractive option in teaching. However, it is of utmost importance to comprehend the implications of this phenomenon. Therefore, conducting studies to gather relevant information on memory and content retention is imperative, as it can assist students in determining the ideal speed^(4)^ and developing effective learning strategies.

Introduced by YouTube in 2010, the video acceleration tool grew exponentially after the pandemic in response to the demand generated by teleworking and telecommunications, among other factors. The primary objective is to optimize time and obtain as much information as necessary. It is important to note that, according to data collected by YouTube, the most frequent time range for using this tool is from 11 pm onwards. In terms of speed preference, analysis indicates that 1.5x speed is the most commonly adopted, followed by 2.0x and 1.25x^(23)^.

The impacts of using this acceleration on the learning process have been studied by several researchers, with different results, depending on the speed of acceleration of reproduction of the audiovisual resource. Studies reports that reproduction speed does not negatively impact memory processing and concentration, therefore not harming learning^(4,12,15,18,20)^. Other researchers add that the usual speed of human speech is 150 words per minute (ppm), while auditory comprehension varies between 150 and 270 words per minute. Above this limit, information needs to be more efficiently captured^(12,15)^. Therefore, 1x, 1.25x, 1.5x, and 1.75x speeds are within this range.

Research has concluded that the best speed for accessing audiovisual resources regarding comprehension and memory retention is between 1.25x and 1.5x^(12)^. On the other hand, the 2.0x speed compromises the ability to retain information, as it overloads the cognitive load on short-term memory^(17)^. Furthermore, in one study, 1x, 1.5x, and 2x were used for quantitative analysis, demonstrating that 1.5x provides more excellent understanding satisfaction than other speeds^(11)^. Therefore, a preference for speeds lower than 2x but higher than average speed is evident.

On the other hand, some scholars contradict the effectiveness of this learning method, arguing that the acceleration of information generates cognitive overload, damaging processing capacity and, probably, more significant fatigue^(16,24)^. Listening involves complex cognitive processes, such as semantic, pragmatic, neurological, and linguistic understanding^(24-26)^.

Variations in accent, unfamiliar subjects, and how the content is presented can make it difficult to absorb the message, requiring more significant cognitive effort on the listener's part. The most crucial difficulty among university students is assimilating content in videos played at high speed, representing 62% of cases. These difficulties include the inability to maintain the sequence of information, form semantic representations, and the limited ability to formulate a mental image of the content presented^(27)^.

Another criticism directed at the acceleration of audiovisual resources refers to the creation of content. We can see in the literature that the absorption of information in short-term memory requires visual elements, such as images, a dynamic and interactive approach, among other facilitators that stimulate concentration and favor the transition to long-term memory^(16)^.

However, some authors argue that speeding up videos can increase concentration on capturing information. The average speed of speech is a topic of significant interest in the academic community. It has been observed that students tend to concentrate on the information within the first 10 to 18 minutes of class, after which their focus gradually diminishes due to fatigue. This suggests that a limited-time use of the acceleration resources should be considered^(12)^. Conversely, other authors have found that accelerated speech can lead to cognitive improvements in tasks that lasted ten minutes at fast speed and nine and a half minutes at slow speed^(14)^. In this study, a faster speed, with an average of 133 words per minute, was associated with a higher intensity, while a slower speed, with an average of 119 words per minute, was associated with a lower intensity.

Video acceleration offers several potential benefits, such as the ease of reviewing content, time optimization, and the ability to enjoy leisure moments^(4)^. However, it's important to note that for new information to be stored in long-term memory, it must pass through working memory, which has a limited storage capacity^(15)^. Therefore, an overload of stimuli in this initial phase can hinder the transfer to subsequent forms of information storage.

In this scoping review, most studies demonstrated good methodological quality. However, there is still a need for studies with greater methodological rigor. It is essential to highlight that the results of subjective studies were limited for a series of reasons, including small samples and the need for adequate control of variables. No studies have compared results in tonal and atonal languages. Furthermore, it is essential to consider the difficulties faced by specific groups, such as individuals with Attention Deficit Hyperactivity Disorder, Autism Spectrum Disorder and learning deficits, second language speakers, to better understand the impacts of making the speed of speech more flexible—reproduction of audiovisual resources about these groups.

While the use of video acceleration has clear advantages in terms of time optimization and access to information, it's crucial to consider the potential impacts on understanding and retention of content. The choice of the ideal speed should not only consider the efficiency in absorbing information, but also the cognitive load involved and the individual characteristics of the target audience. Therefore, it's imperative that future studies focus on further investigating these issues, with the aim of providing more accurate and effective guidance for using this tool in the educational context and beyond.

This scoping review provides a crucial reflection on optimizing time through accelerating speech and its implications. It is essential that the population, especially university students, benefit from different reproduction speeds appropriately, and it is also the responsibility of educational institutions to guide students so that use is more effective and healthy.

It is essential to recognize some limitations, such as the limited diversity of participants, the lack of adaptation of tests to individual needs, unfavorable conditions in the study environment, and the use of isolated words to assess the impact of speed, which is very far from actual use. of this tool. Therefore, it is essential that future research expand the sample, consider the diversity of accents, and provide greater autonomy to participants in controlling speed to understand the difficulties each faces better. Furthermore, specific tests that assess memorization and comprehension capacity with diverse answer options are necessary, as is the description in all studies of the number of words per minute and the task execution time.

It is important to highlight that a scoping review does not address the relative weight of evidence in favor of the effectiveness of the criticized interventions but rather a narrative or descriptive report of the available research. This review will help the population choose the ideal reproduction speed, with an understanding of its impacts and regulation according to learning interests, effectively and satisfactorily, without compromising the assimilation of the content. However, new studies are necessary, as the number of studies is still small. There needs to be more consensus in the literature regarding the influence of speech rate on the learning process and homogeneity in the design of the studies aiming at the generalization of these findings.

## CONCLUSION

It was observed that reproduction speed rates of audiovisual resources impact university students' learning process. Rates greater than 2x limit the learning process. Rates within the normal range (between 1x and 1.75x) are favorable for capturing the content differences and optimize playback speeds for different learning contexts.

## Appendix A Search strategy

**Table:**

**Database**	**Search (september 13, 2023)**	**Search (september 13 2023 - march 08, 2025)**
SciElo	0 ((("Undergraduate student" OR "Undergraduate students" OR "Undergraduate" OR "Undergraduates" OR University OR Universities) AND "speech rate" OR "speed watching" OR "playback speed" OR "high-speed video" OR "video speed" OR "video playback speed" OR "video playback-speed" OR "accelerated playback" OR "video lecture" OR "video-recorded lecture") AND (memory OR memories OR repetition OR "learning" OR "oral comprehension" OR "oral language comprehension" OR "processing speed" OR comprehension)))	0 ((("Undergraduate student" OR "Undergraduate students" OR "Undergraduate" OR "Undergraduates" OR University OR Universities) AND "speech rate" OR "speed watching" OR "playback speed" OR "high-speed video" OR "video speed" OR "video playback speed" OR "video playback-speed" OR "accelerated playback" OR "video lecture" OR "video-recorded lecture") AND (memory OR memories OR repetition OR "learning" OR "oral comprehension" OR "oral language comprehension" OR "processing speed" OR comprehension)))
Lilacs	3 (("Undergraduate student" OR "Undergraduate students" OR "Undergraduate" OR "Undergraduates" OR University OR Universities) ) AND (("speech rate" OR "speed watching" OR "playback speed" OR "high-speed video" OR "video speed" OR "video playback speed" OR "video playback-speed" OR "accelerated playback" OR "video lecture" OR "video-recorded lecture")) AND ((memory OR memories OR repetition OR "learning" OR "oral comprehension" OR "oral language comprehension" OR "processing speed" OR comprehension))	3 (("Undergraduate student" OR "Undergraduate students" OR "Undergraduate" OR "Undergraduates" OR University OR Universities) ) AND (("speech rate" OR "speed watching" OR "playback speed" OR "high-speed video" OR "video speed" OR "video playback speed" OR "video playback-speed" OR "accelerated playback" OR "video lecture" OR "video-recorded lecture")) AND ((memory OR memories OR repetition OR "learning" OR "oral comprehension" OR "oral language comprehension" OR "processing speed" OR comprehension))
PubMed	477 ((("Undergraduate student"[All Fields] OR "Undergraduate students"[All Fields] OR "Undergraduate"[All Fields] OR "Undergraduates"[All Fields] OR ("universiti"[All Fields] OR "universities"[MeSH Terms] OR "universities"[All Fields] OR "university"[All Fields] OR "university s"[All Fields]) OR ("universiti"[All Fields] OR "universities"[MeSH Terms] OR "universities"[All Fields] OR "university"[All Fields] OR "university s"[All Fields])) AND "speech rate"[All Fields]) OR (("speed"[All Fields] OR "speeded"[All Fields] OR "speeding"[All Fields] OR "speeds"[All Fields]) AND ("watch"[All Fields] OR "watched"[All Fields] OR "watches"[All Fields] OR "watching"[All Fields])) OR "playback speed"[All Fields] OR "high-speed video"[All Fields] OR "video speed"[All Fields] OR "video playback-speed"[All Fields] OR "video playback-speed"[All Fields] OR "accelerated playback"[All Fields] OR "video lecture"[All Fields] OR "video-recorded lecture"[All Fields]) AND ("memories"[All Fields] OR "memory"[MeSH Terms] OR "memory"[All Fields] OR "memory s"[All Fields] OR ("memories"[All Fields] OR "memory"[MeSH Terms] OR "memory"[All Fields] OR "memory s"[All Fields]) OR ("repetition"[All Fields] OR "repetitions"[All Fields]) OR "learning"[All Fields] OR "oral comprehension"[All Fields] OR "oral language comprehension"[All Fields] OR "processing speed"[All Fields] OR ("comprehensibility"[All Fields] OR "comprehensible"[All Fields] OR "comprehension"[MeSH Terms] OR "comprehension"[All Fields] OR "comprehensions"[All Fields] OR "comprehensive"[All Fields] OR "comprehensively"[All Fields] OR "comprehensiveness"[All Fields]))	83 (((("Undergraduate student"[All Fields] OR "Undergraduate students"[All Fields] OR "Undergraduate"[All Fields] OR "Undergraduates"[All Fields] OR ("universiti"[All Fields] OR "universities"[MeSH Terms] OR "universities"[All Fields] OR "university"[All Fields] OR "university s"[All Fields]) OR ("universiti"[All Fields] OR "universities"[MeSH Terms] OR "universities"[All Fields] OR "university"[All Fields] OR "university s"[All Fields])) AND "speech rate"[All Fields]) OR (("speed"[All Fields] OR "speeded"[All Fields] OR "speeding"[All Fields] OR "speeds"[All Fields]) AND ("watch"[All Fields] OR "watched"[All Fields] OR "watches"[All Fields] OR "watching"[All Fields])) OR "playback speed"[All Fields] OR "high-speed video"[All Fields] OR "video speed"[All Fields] OR "video playback-speed"[All Fields] OR "video playback-speed"[All Fields] OR "accelerated playback"[All Fields] OR "video lecture"[All Fields] OR "video-recorded lecture"[All Fields]) AND ("memories"[All Fields] OR "memory"[MeSH Terms] OR "memory"[All Fields] OR "memory s"[All Fields] OR ("memories"[All Fields] OR "memory"[MeSH Terms] OR "memory"[All Fields] OR "memory s"[All Fields]) OR ("repetition"[All Fields] OR "repetitions"[All Fields]) OR "learning"[All Fields] OR "oral comprehension"[All Fields] OR "oral language comprehension"[All Fields] OR "processing speed"[All Fields] OR ("comprehensibility"[All Fields] OR "comprehensible"[All Fields] OR "comprehension"[MeSH Terms] OR "comprehension"[All Fields] OR "comprehensions"[All Fields] OR "comprehensive"[All Fields] OR "comprehensively"[All Fields] OR "comprehensiveness"[All Fields]))) AND (2023/9/10:2025/3/8[pdat])
Scopus	459 TITLE-ABS-KEY ( ( ( "Undergraduate student" OR "Undergraduate students" OR "Undergraduate" OR "Undergraduates" OR university OR universities ) AND "speech rate" OR "speed watching" OR "playback speed" OR "high-speed video" OR "video speed" OR "video playback speed" OR "video playback-speed" OR "accelerated playback" OR "video lecture" OR "video-recorded lecture" ) AND ( memory OR memories OR repetition OR "learning" OR "oral comprehension" OR "oral language comprehension" OR "processing speed" OR comprehension ) )	116 TITLE-ABS-KEY ( ( ( "Undergraduate student" OR "Undergraduate students" OR "Undergraduate" OR "Undergraduates" OR university OR universities ) AND "speech rate" OR "speed watching" OR "playback speed" OR "high-speed video" OR "video speed" OR "video playback speed" OR "video playback-speed" OR "accelerated playback" OR "video lecture" OR "video-recorded lecture" ) AND ( memory OR memories OR repetition OR "learning" OR "oral comprehension" OR "oral language comprehension" OR "processing speed" OR comprehension ) ) AND PUBYEAR > 2022 AND PUBYEAR < 2026
Web Of Science	1113 (("Undergraduate student" OR "Undergraduate students" OR "Undergraduate" OR "Undergraduates" OR University OR Universities) AND "speech rate" OR "speed watching" OR "playback speed" OR "high-speed video" OR "video speed" OR "video playback speed" OR "video playback-speed" OR "accelerated playback" OR "video lecture" OR "video-recorded lecture") AND (memory OR memories OR repetition OR "learning" OR "oral comprehension" OR "oral language comprehension" OR "processing speed" OR comprehension) (All Fields)	228 (("Undergraduate student" OR "Undergraduate students" OR "Undergraduate" OR "Undergraduates" OR University OR Universities) AND "speech rate" OR "speed watching" OR "playback speed" OR "high-speed video" OR "video speed" OR "video playback speed" OR "video playback-speed" OR "accelerated playback" OR "video lecture" OR "video-recorded lecture") AND (memory OR memories OR repetition OR "learning" OR "oral comprehension" OR "oral language comprehension" OR "processing speed" OR comprehension) (All Fields) and 2023 or 2024 or 2025 (Publication Years)
Google Scholar	100 (("Undergraduate student" OR "Undergraduate students" OR "Undergraduate" OR "Undergraduates" OR University OR Universities) ) AND (("speech rate" OR "speed watching" OR "playback speed" OR "high-speed video" OR "video speed" OR "video playback speed" OR "video playback-speed" OR "accelerated playback" OR "video lecture" OR "video-recorded lecture")) AND ((memory OR memories OR repetition OR "learning" OR "oral comprehension" OR "oral language comprehension" OR "processing speed" OR comprehension))	100 (("Undergraduate student" OR "Undergraduate students" OR "Undergraduate" OR "Undergraduates" OR University OR Universities) ) AND (("speech rate" OR "speed watching" OR "playback speed" OR "high-speed video" OR "video speed" OR "video playback speed" OR "video playback-speed" OR "accelerated playback" OR "video lecture" OR "video-recorded lecture")) AND ((memory OR memories OR repetition OR "learning" OR "oral comprehension" OR "oral language comprehension" OR "processing speed" OR comprehension))
